# Morphogenesis of the anterior segment in the zebrafish eye

**DOI:** 10.1186/1471-213X-5-12

**Published:** 2005-06-28

**Authors:** Kelly A Soules, Brian A Link

**Affiliations:** 1 Department of Cell Biology, Neurobiology and Anatomy, Medical College of Wisconsin, Milwaukee, Wisconsin, USA

## Abstract

**Background:**

The ocular anterior segment is critical for focusing incoming light onto the neural retina and for regulating intraocular pressure. It is comprised of the cornea, lens, iris, ciliary body, and highly specialized tissue at the iridocorneal angle. During development, cells from diverse embryonic lineages interact to form the anterior segment. Abnormal migration, proliferation, differentiation, or survival of these cells contribute to diseases of the anterior segment such as corneal dystrophy, lens cataract, and glaucoma. Zebrafish represent a powerful model organism for investigating the genetics and cell biology of development and disease. To lay the foundation for genetic studies of anterior segment development, we have described the morphogenesis of this structure in zebrafish.

**Results:**

As in other vertebrates, the zebrafish anterior segment derives from diverse origins including surface ectoderm, periocular mesenchyme, and neuroepithelium. Similarly, the relative timing of tissue differentiation in the anterior segment is also conserved with other vertebrates. However, several morphogenic features of the zebrafish anterior segment differ with those of higher vertebrates. These include lens delamination as opposed to invagination, lack of iris muscles and ciliary folds, and altered organization in the iridocorneal angle. In addition, substantial dorsal-ventral differences exist within the zebrafish anterior segment.

**Conclusion:**

Cumulatively, our anatomical findings provide a reference point to utilize zebrafish for genetic studies into the mechanisms of development and maintenance of the anterior segment.

## Background

The anterior segment of the vertebrate eye is comprised of the cornea, lens, iris, ciliary body, and highly specialized tissue at the iridocorneal angle. Two main functions are ascribed to the ocular anterior segment. The first is to focus incoming light onto the neural retina and the second is to regulate intraocular pressure. For mammals and other higher vertebrates, refraction of light entering the eye is accomplished by both the transparent cornea and lens. In many aquatic vertebrates, including fish, the lens is solely responsible for focusing incoming light [1,2]. In all vertebrates, intraocular pressure is maintained by the balance between aqueous humor production and outflow [3]. The dynamics of aqueous humor have been best characterized in mammals where ciliary epithelial cells produce the clear ocular fluid while the trabecular meshwork, which is situated at the iridocorneal angle overlying Schlemm's canal, regulates drainage.

The structures of the anterior segment arise from diverse embryonic lineages and there is exquisite coordination among the different compartments during development. Studies in avian and mammalian species have shown that tissues of the anterior segment derive from surface ectoderm, head mesoderm, neural crest and neuroectoderm [4-7]. Development of the anterior segment initiates with the invagination of the lens from surface ectoderm. With establishment of the lens vesicle, head mesoderm and neural crest cells migrate into a periocular location and eventually move into the anterior segment of the rudimentary eye between the surface ectoderm and the neural retina and lens. These mesenchymal cells differentiate into the corneal endoderm, structures at the iridocorneal angle, and iris and ciliary body stroma. The non-pigmented and pigmented epithelium of the iris and ciliary epithelium derive from the peripheral edge of the retinal neuroepithelium and retinal pigmented epithelium, respectively. Developmental anatomy of the anterior segment for many higher vertebrates has been well characterized and excellent reviews exist [8,9]. However, the relevant cellular interactions between various structures of the anterior segment and the molecular basis of development is just beginning to be understood.

A detailed understanding of the mechanisms of development of the anterior segment can provide general insights into questions such as tissue induction, cell type fate determination, and the regulation of cellular morphogenesis. In addition, an understanding of ontogeny of the anterior segment has significance to several human diseases. Primarily, several forms of glaucoma are associated with anterior segment dysgenesis and genes which are essential for formation of this part of the eye can promote glaucoma [9,10]. Corneal dystrophies and lens cataracts are additional examples of anterior segment disease. The zebrafish has many experimental advantages for studying both development and disease phenotypes, including those for glaucoma [11]. These include the ability to conduct genetic screens for complex traits owing to high fecundity and genomic infrastructure. In addition, ease of transgenesis for target gene functional analysis, coupled with rapid and transparent initial development, facilitates cell behavior characterization via time-lapse microscopy. However, an overview of the development for the zebrafish anterior segment has not been described and the extrapolation of ocular anatomy from other teleost species is not favorable due the high morphological diversification of the bony fish [1]. In this study we report the characterization by light and transmission electron microscopy (TEM) the morphogenesis of the zebrafish anterior segment. We find that while there is rapid initial establishment of the anterior segment, occurring within the first three days of embryogenesis, significant growth and morphogenesis continues until approximately 1 month when the mature morphology is attained. There are also significant differences in the elaboration of dorsal versus ventral regions within the zebrafish anterior segment. Importantly, both similarities and differences exist between the anatomy of the zebrafish ocular anterior segment and that of mammalian eyes.

## Results

### Establishment of the anterior chamber

**Figure 1:**
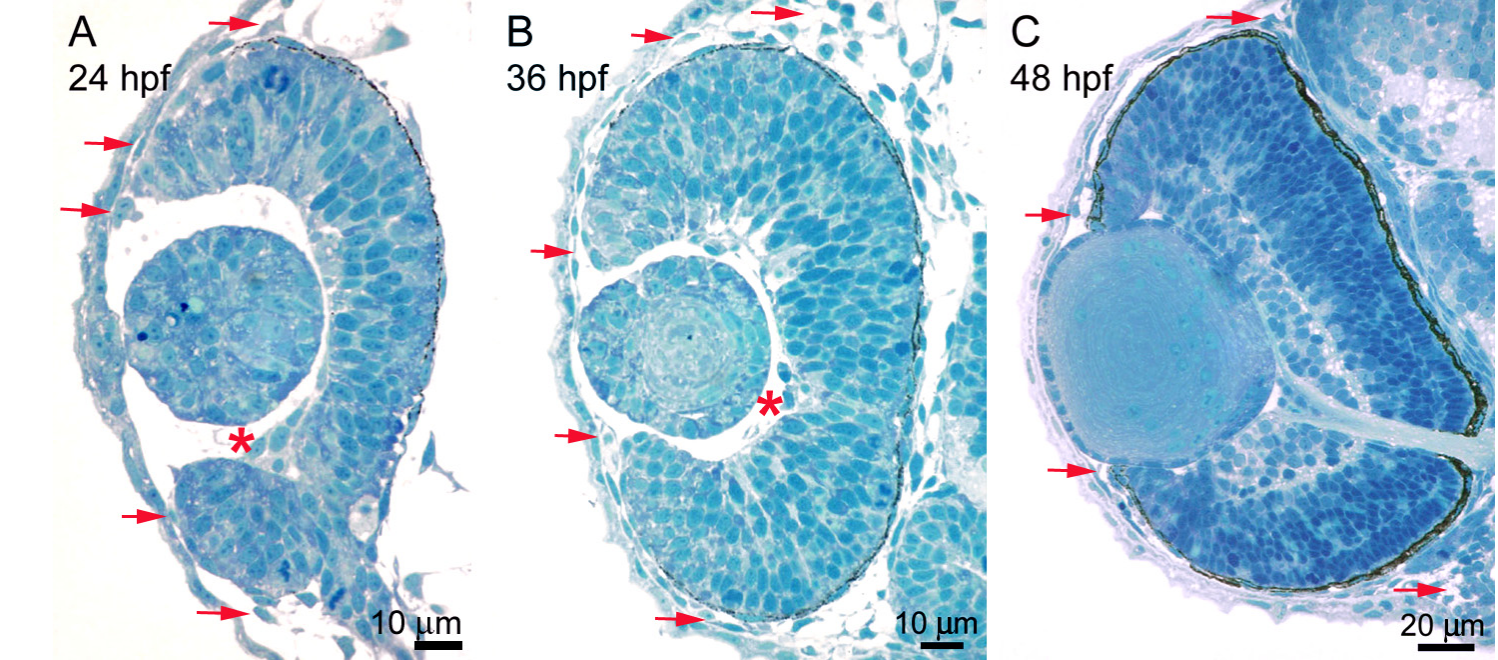
**Establishment of the anterior chamber. **Histology of a 24 hpf (A), 36 hpf (B), and 48 hpf (C) zebrafish eye. Periocular mesenchyme is present at 24 hpf, but more prominent at 36 and 48 hpf (arrows). Hyaloid vasculature is indicated with asterisk.

**Figure 2:**
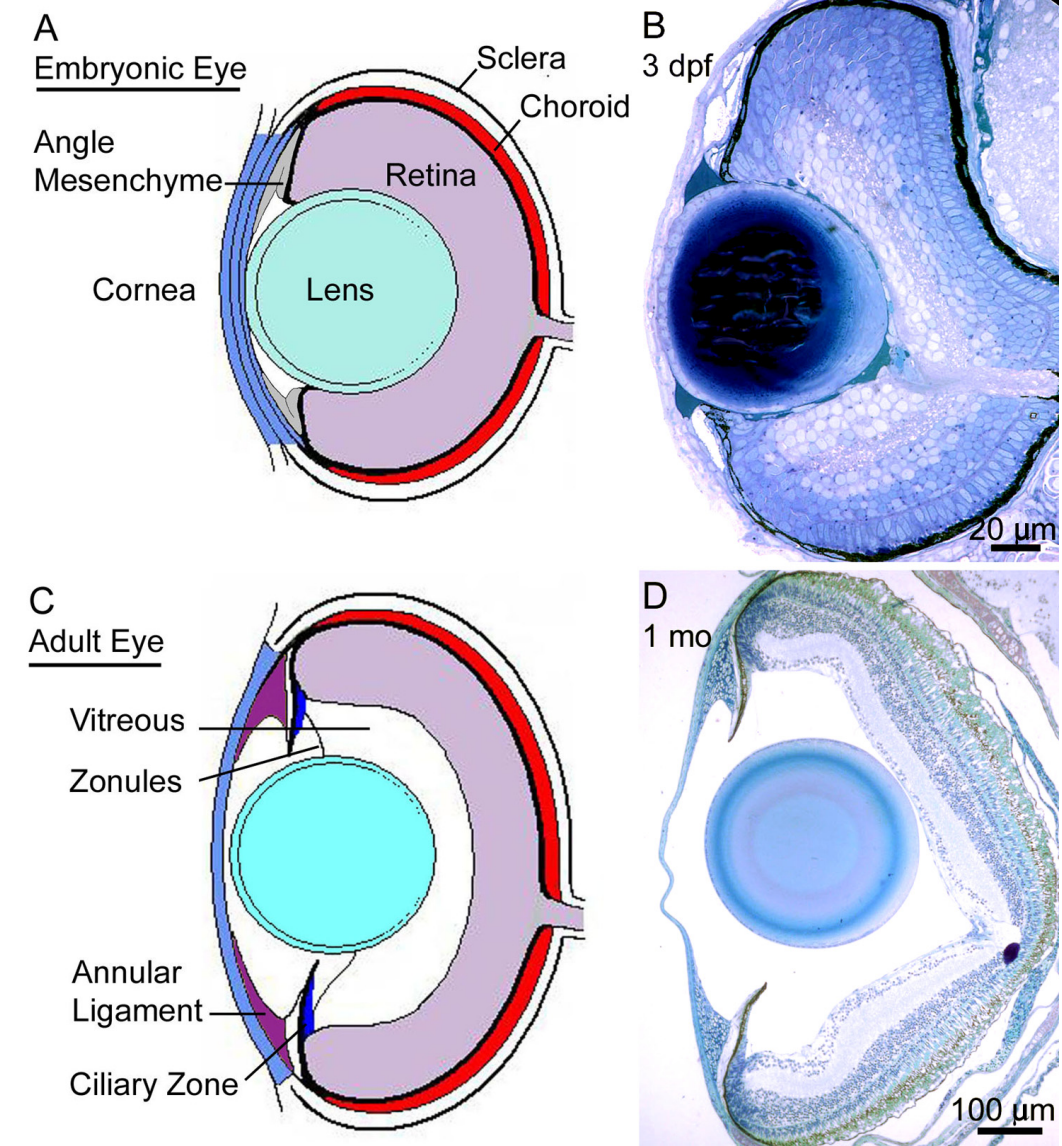
**Comparison of embryonic and adult zebrafish eyes. **Diagram of embryonic (A) and adult (C) zebrafish eyes. Histology of 3 dpf embryonic (B) and 1 month adult (D) eyes.

The zebrafish anterior segment is established very rapidly and rudimentary structures of the anterior segment are present by 3 days post fertilization (dpf) when visually evoked behaviors are first observed [12]. The anterior chamber forms with the detachment of the lens vesicle from the surface ectoderm which occurs by 26 hours post fertilization (hpf). Beginning at approximately 24 hpf, mesenchymal cells migrate from periocular locations into the anterior segment (Figure 1; [12,13]). Within the anterior segment, a single layer of flattened mesenchymal cells associated with the posterior portion of the cornea can be seen by 36 hpf (Figure 1B). At this time within the peripheral angles of the anterior chamber, undifferentiated mesenchymal cells accumulate. Hyaloid vasculature is associated with the posterior region of the lens and ciliary vessels, which circumvent the anterior rim of the eye, and enter and exit at the embryonic fissure (Figure 1A, B). By 48 hpf, differentiation among mesenchymal cells can be detected within the angle region where the cornea and prospective iris meet (Figure 1C). By 3 dpf, multiple types of pigment cells, as well as less differentiated non-pigmented cells, are present at the angle (Figure 2A, B). Tissue extension from the margins of the neural retina has established the iris anlagen. The ellipsoid shape of the eye and large spherical lens make the newly formed anterior chamber relatively shallow at the center as compared to the periphery. While the rudimentary anterior segment is formed by day 3, extensive growth and morphogenesis occurs until 1 month when the eye reaches its mature form (Figure 2). Specifically there is extensive stratification within the cornea, differentiation and morphogenesis of the iris stroma and ciliary epithelial zones, specialization of angle structures, and elaboration of dorsal versus ventral differences. Interestingly, following initial establishment of the anterior segment, growth and morphogenesis appear to be coupled and independent of the age of the fish. For example, 17 dpf sibling zebrafish reared under the same conditions in the same tank can have measurable body length differences. Both eye size and anterior segment differentiation correlate with body length, but not the absolute age of the fish (data not show). For this reason, body length measurements were recorded for each specimen examined. Following 1 month, continued eye growth and subtle refinement of the zebrafish anterior segment persists past sexual maturity (~ 3 months).

### The cornea

**Figure 3:**
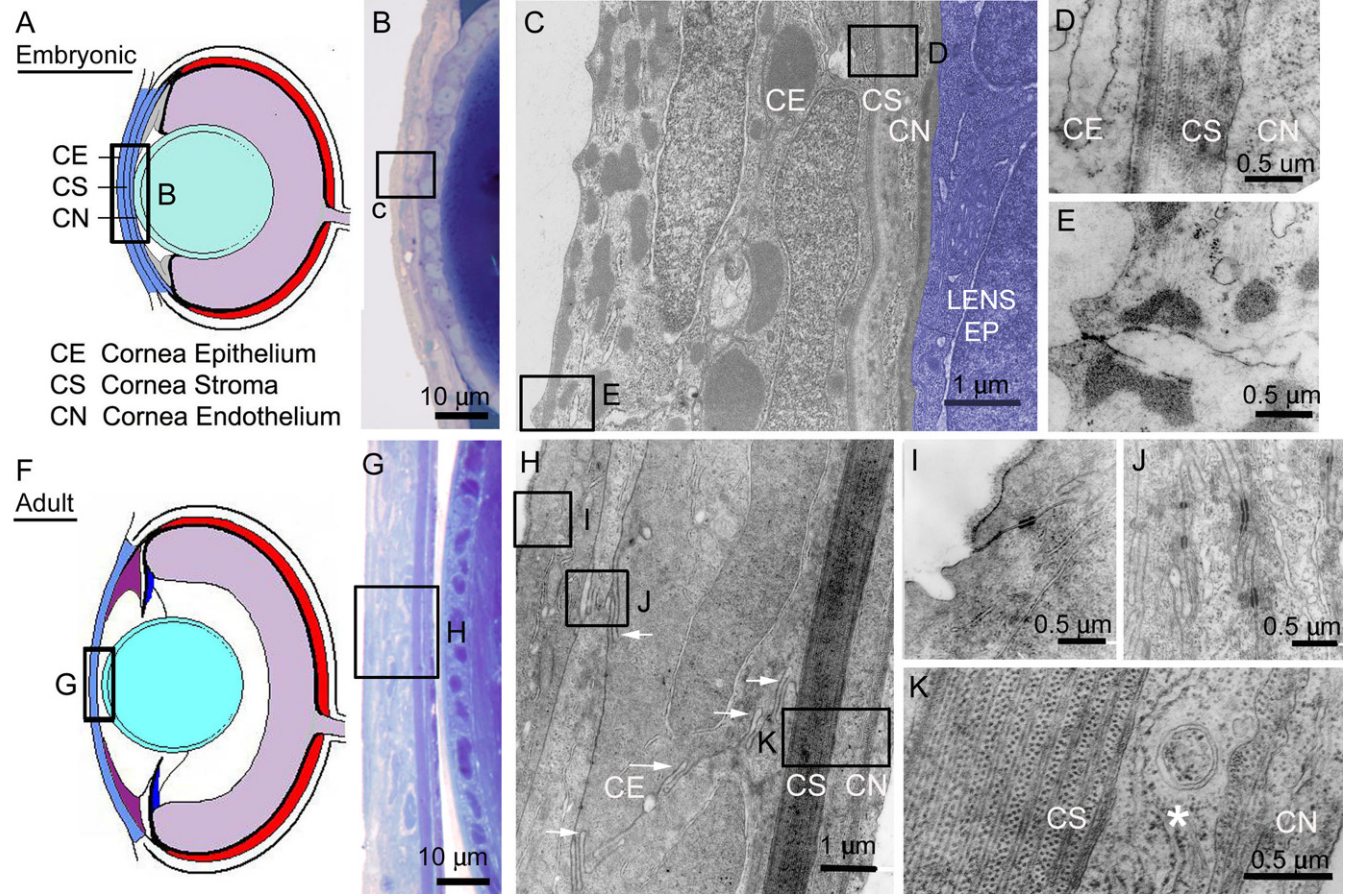
**Cornea morphogenesis. **Diagram of embryonic (A) and adult (F) zebrafish corneas. Morphology of embryonic (B-E) and adult corneas (G-K). Histology of cornea and anterior lens of 3 dpf embryo (B). TEM micrograph showing embryonic cornea epithelium, CE; cornea stroma, CS; and cornea endothelium, CN (C). In this panel, the lens epithelium (Lens EP) is indicated by blue shading. High magnification insets of developing cornea stroma (D) and "scalloped" cornea epithelium of 3 dpf embryo (E). Histology of cornea and anterior lens of 1 month adult (G). TEM micrograph showing 1 month cornea epithelium, cornea stroma, and cornea endothelium (H). Extensive interdigitation between epithelial cells is indicated with arrows (H). High magnification insets of the mature cornea epithelial cells (I), cornea sub-epithelial cells (J), and cornea stroma and endothelium (K). Note the increase of electron dense cell junctions in cornea sub-epithelial cells (J) and the establishment of a flattened layer of cells, indicated with asterisk, between the stroma and endothelium (K). In both the developing and mature cornea stroma, orthogonally arranged collagen fibrils are clearly visible and the stromal thickness has increased with development (D and K).

In all vertebrates studied, the cornea develops from both the surface ectoderm and periocular mesenchyme. Following detachment of the lens vesicle from the surface ectoderm, mesenchymal cells migrate into the anterior chamber along the surface ectoderm. The surface ectoderm and periocular mesenchyme differentiate into the corneal epithelium and endothelium, respectively. In mammals and avians, the corneal stroma is then formed from an additional wave of immigrating periocular mesenchymal cells [14]. The corneal stroma further stratifies through cellular differentiation and the deposition of highly ordered collagen fibrils and other extracellular matrix components. Histological observations support a similar mode of corneal establishment in zebrafish (Figure 1). Up to 60 hpf, the zebrafish cornea consists of relatively undifferentiated, multi-stratified cells, although distinct layers are not obvious. Differentiation within the cornea, however, is readily observable by 3 dpf as stratification becomes apparent (Figure 3). At this time, the surface epithelium, which is still continuous with the skin of the embryo, is composed of hexagonally shaped cells that appear scalloped is cross-section. Electron microscopy revealed the presence of numerous electron dense adherens junctions between surface epithelial cells, but few such cell junctions were found between the 2–3 layers of subepithelial cells (Figure 3C, E). During development, the corneal surface epithelium can also be distinguished ultrastructurally from subepithelial cells by the type of inclusion bodies. Surface epithelium shows numerous dark-staining small inclusion bodies, while subepithelia contain less numerous, but larger inclusion bodies (Figure 3C). A thin lamellar stroma with orthogonally arrayed collagen fibrils is visible by 3 days (Figure 3D). Within the periphery, the corneal stroma is continuous with the sclera. In the 3–5 dpf embryo, the thin collagen stroma directly overlays flattened endothelial cells (Figure 3C).

By one month, the cornea has further stratified (Figure 3G, H). The epithelium has lost its scalloped appearance and is no longer continuous with the outer epidermis. As compared to mammals, the corneal epithelium is relatively thick containing 3–4 layers of interdigitated cells (arrows, Figure 3H). An increase in the number of adherens juctions was noted between surface epithelial cells and subepithelial cells (Figure 3I, J). A thin extracellular deposition (Bowman's membrane) separates the subepithelial cells from the the corneal stroma layer. The stroma, although thin compared to mammals, has increased in thickness with development and maintains the orthogonal array of collagen fibrils (Figure 3K). Sparsely distributed, flattened cells can be seen upon electron microscopy within the stroma, particularly at the peripheral edges. The number of these keratocytes, as well as the thickness of the stroma, increases with age past 1 month. At the peripheral limbal region, the stroma splits into multiple layers. Just beneath the stroma is a single layer of cells overlying a second, thinner stromal layer with similar collagen organization (Figure 3K, [15]). Subjacent to these cells, an additional collagen-rich extracellular layer can be seen with electron microscopy (Descemet's membrane). The endothelium is also thin compared to mammals and is comprised of a single layer of flattened cells which extend over the iridocorneal angle covering the surface of the annular ligament. Mucus secreting goblet cells were frequently observed at the peripheral edges of the cornea (data not shown).

### The lens

**Figure 4:**
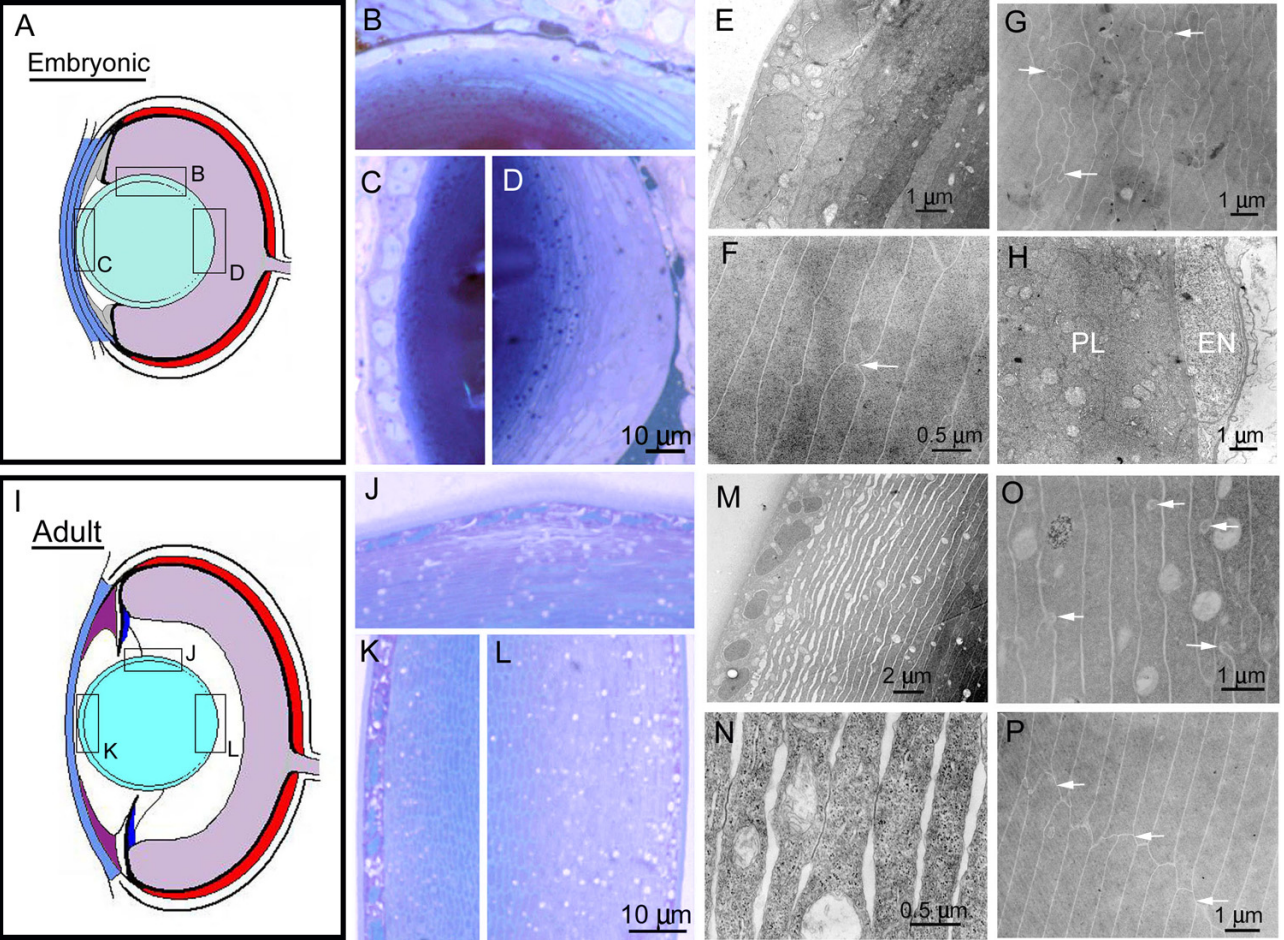
**Lens morphogenesis**. Diagram of the embryonic (A) and adult (I) zebrafish lens. Insets in (A) indicate corresponding histological sections through the 3 dpf lens transition zone (B), lens epithelium (C) and posterior lens (D). TEM micrographs (E-H) show developing lens epithelium (E), interdigitating lens fiber cells (arrows, G), establishment of rudimentary lens sutures (arrow, F), and a vascular endothelial cell, EN, associated with the posterior lens, PL (H). Insets in (I) indicate corresponding histological sections through the 1 month lens transition zone (J), lens epithelium (K) and posterior lens (L). TEM micrographs (M-P) show the mature lens epithelium (M), "ball and socket" interdigitations between fiber cells (arrows, O), loose fiber cell organization at the periphery (N), and mature suture connections between the ends of lens fiber cells (arrows, P).

The zebrafish lens is relatively large and spherical, unlike mammalian and avian lenses which are more ellipsoid in shape. The zebrafish lens shows typical vertebrate composition with two cell types-lens epithelial cells and lens fiber cells. The establishment of the lens begins with the contact between the evaginating optic vesicle and surface ectoderm at 16 hpf (14–15 somite stage). This interaction results in a visible thickening of the lens placode [13]. Unlike the mammal, the lens of the zebrafish forms by delamination of lens placodal cells and not through invagination. This results in a solid spherical mass as opposed to a hollow lens vesicle (Figure 1A; [12,13]). Detachment of the solid lens vesicle of zebrafish at 24–26 hpf is accomplished in part by apoptosis, similar to mammals [16]. By 30 hpf, lens epithelial cells are discernible. Primary fiber cell elongation occurs in a circular fashion within the center of the newly formed lens. A transition zone is present at 36 hpf and is located more posteriorly as compared to mammals. This region of the lens is common among vertebrates and is where secondary fibers cells differentiate from proliferative epithelial cells. Loss of organelles within lens fiber cells is apparent by 72 hpf. We have focused our analysis on three diverse regions of the lens: the anterior epithelium, the transition zone, and the posterior lens (Figure 4). In the developing lens, anterior epithelial cells are cuboidal with flat plasma membranes (Figure 4E). Some interdigitations are present laterally between these cells. Large, irregularly shaped nuclei reside in the center of the lens epithelial cytoplasm. A thin extracellular capsule is present by 72 hpf. At the transition zone, epithelial nuclei elongate with the lengthening and differentiation into fiber cells (Figure 4B). The more centrally located, differentiated fiber cells show dark staining intracellular spheres, perhaps related to the disassembly of organelles. These dark staining bodies are also observed within the posterior lens (Figure 4D). Newly generated fiber cells show extensive interdigitations, but have yet to achieve either "ball and socket" or suture organization (Figure 4G, F) At this stage of development, very little vitreous exists and the lens is often observed in direct contact with ganglion cells of the neural retina as well as vascular hyaloid cells (Figure 4H).

**Figure 5:**
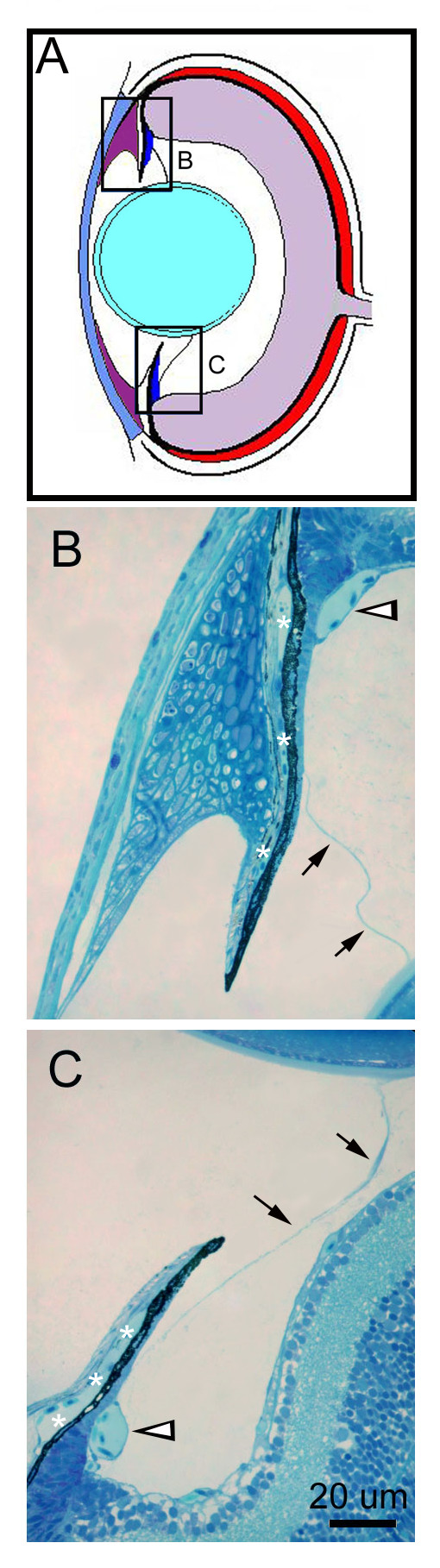
**Lens zonules. **Diagram for location of mature lens zonules (A). Histological sections through the dorsal (B) and ventral (C) anterior segment. Lens zonules are indicated by black arrows. Large ciliary circumferential arteries are indicated with white arrowheads. Iris stroma vasculature in indicated with asterisks.

The lens of the one month old zebrafish retains spherical symmetry. The anterior epithelial cells and their nuclei appear more flattened than at younger ages (Figure 4K and 4M). The capsule is now more thick and prominent. The anterior epithelium covers lens fiber cells which appear hexagonal in cross-section. The packing density of the fiber cells increase in a peripheral to central manner. At the peripheral edges, fiber cells frequently show separations following histological processing due to the lower packing density (Figure 4M, N). Centrally located fiber cells now show "ball and socket" intracellular connections (Figure 4O). At the posterior lens, fiber cell ends meet to form sutures (Figure 4P). At one month, the lens is suspended by zonules attaching the lens capsule at the equator to the non-pigmented epithelium of the ciliary zone (Figure 5). The zonules appear as modified or "toughened" vitreous, similar to those described for other lower vertebrates [1]. In zebrafish, dorsal zonules are thickened as compared to ventral zonules. In both dorsal and ventral regions, these fibers delimit the vitreous body-aqueous humor boundary.

### The iris and ciliary zone

**Figure 6:**
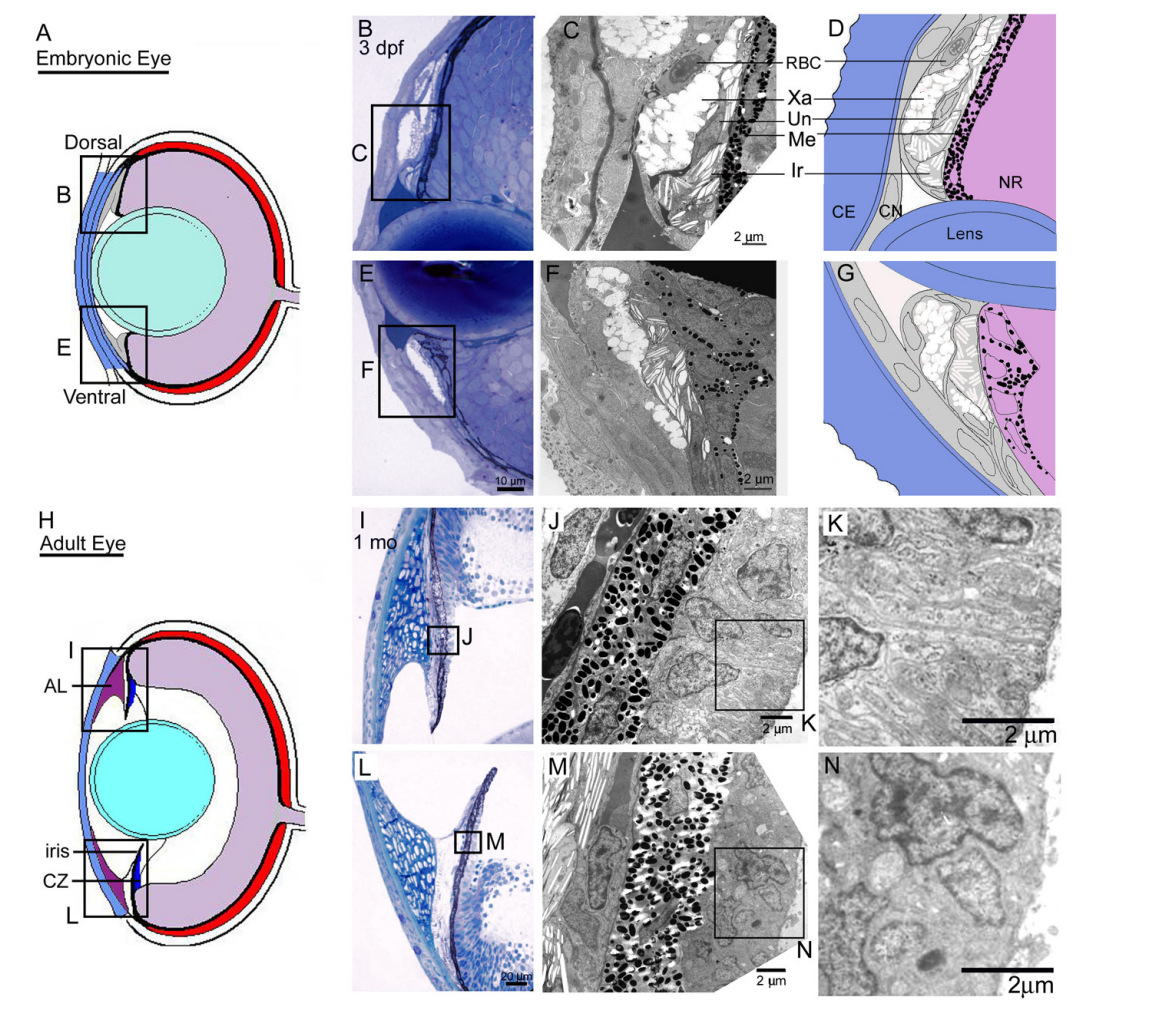
**Morphogenesis of the iris and ciliary zone. **Diagram showing location of insets for the developing (A) and mature (H) iris and ciliary zone. Histology of the 3 dpf developing dorsal (B) and ventral (E) iris. TEM micrographs from 3 dpf show pigment cell stratification in dorsal (C) and ventral (F) regions. Note the reduced differentiation as indicated by pigmentation within the ventral region. Corresponding drawings indicate the principle cell types found within the developing iris (D, G). Color shadings indicate cells of similar embryonic origins. Histology of the 1 month old zebrafish dorsal (I) and ventral (L) iris. TEM micrographs show differences in the ultrastructure of non-pigmented ciliary epithelial cells between dorsal (J) and ventral (M) regions. Insets show the membranous infolding and secretory appearance within the dorsal ciliary zone (K) and the lack of this morphology in the ventral non-pigmented ciliary epithelium (N). RBC, red blood cell; UN, undifferentiated cell; Xa, xanthophore; Me, melanocyte; Ir, iridophore; CE, corneal epithelium; CN, corneal endothelium; NR, neural retina.

In zebrafish, the iris, ciliary epithelium and anterior segment angle specializations differentiate relatively late and in a more protracted fashion as compared to the cornea and lens. The iris and ciliary zone develop from the anterior margin of the retinal neuroepithelium as in other vertebrates. Iris stroma is derived from periocular mesenchyme. Morphological differentiation within the iris stroma is apparent by 3 dpf (Figure 6). Ordered layering of pigment cells within this iris stroma is visible at this early time (Figure 6C, F). The pigment epithelium contains dense, black melanosomes. At 3 dpf, the non-pigmented epithelial cell layer (ciliary or iris) is absent, as retinal margin neuroepithelial cells have yet to differentiate anteriorly. Iridophores, containing rod-shaped iridosome organelles are found directly overlying the pigment epithelial layer. Adjacent and superficial to iridophores are xanthopohores. These pigment cells contain foamy appearing pteranosome organelles. In whole view, iridophores appear silver in color, while xanthophores are gold. Interspersed within the developing iris stroma are non-pigmented, undifferentiated mesenchymal cells, and developing vasculature. In the ventral iris, differentiation of pigment cells appears delayed compared to the dorsal iris as both melanosomes and pteranosomes are reduced in number (Figure 6B–G).

**Figure 7:**
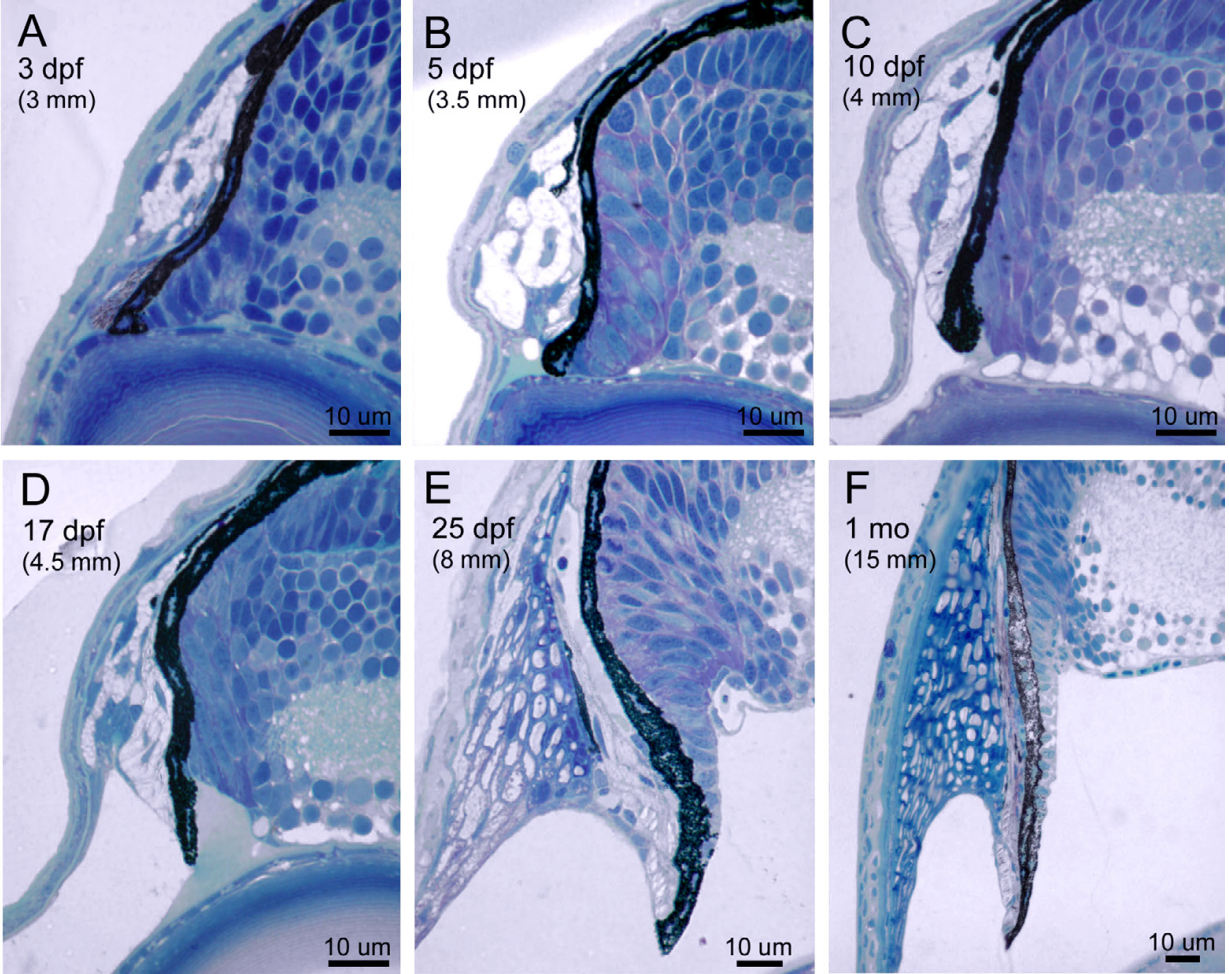
**Morphogenesis of the dorsal iridocorneal angle. **Histological sections through the dorsal iridocorneal angle region of 3 dpf (A), 5 dpf (B), 10 dpf (C), 17 dpf (D), 25 dpf (E) and 1 month (F) zebrafish. Body length is indicated in parenthesis (upper left of each image) as differentiation in the iridocorneal angle is more dependant on the size of the fish than the absolute age.

**Figure 8:**
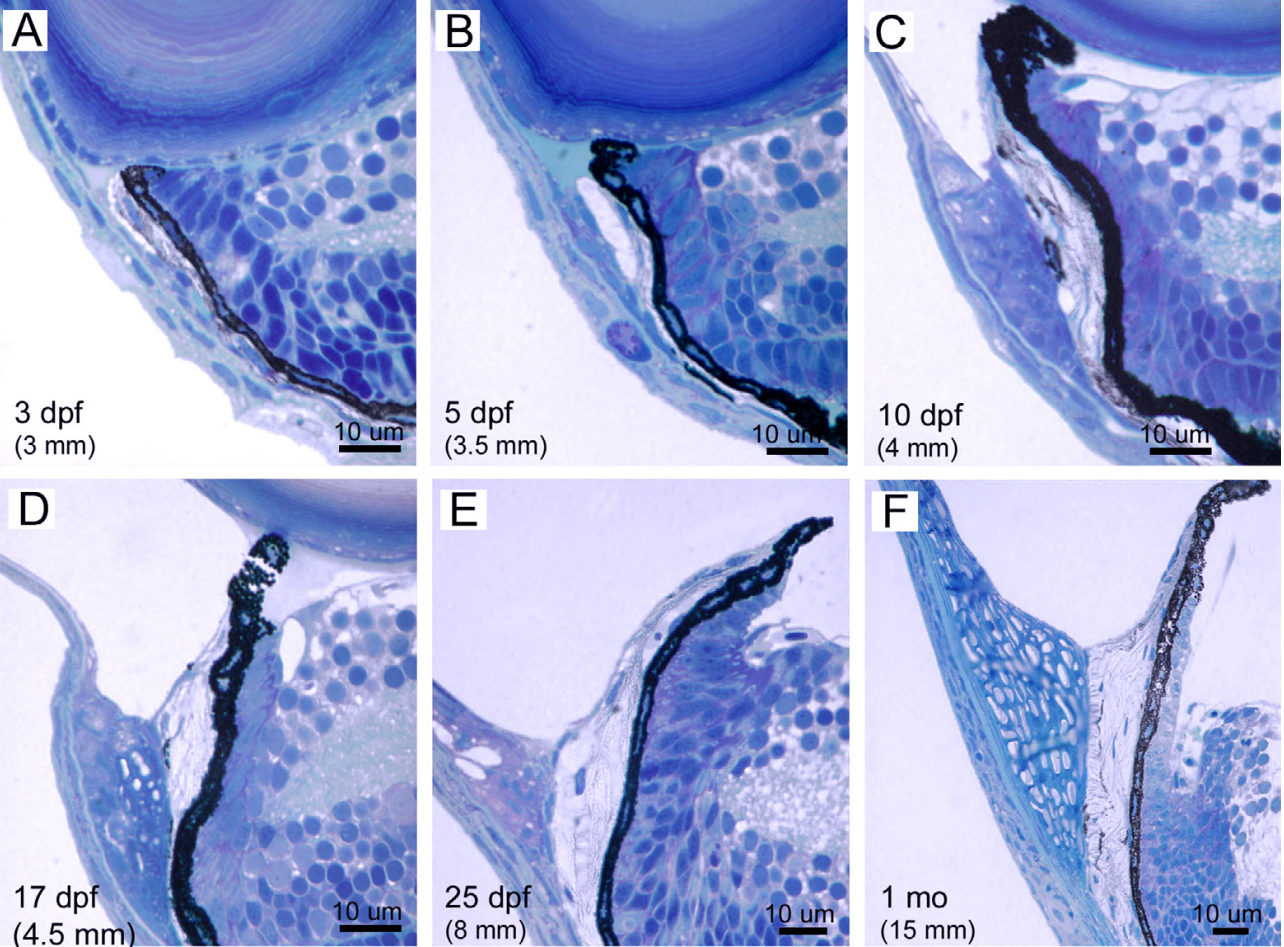
**Morphogenesis of the ventral iridocorneal angle. **Histological sections through the ventral iridocorneal angle region of 3 dpf (A), 5 dpf (B), 10 dpf (C), 17 dpf (D), 25 dpf (E) and 1 month (F) zebrafish. Body length is indicated in parenthesis (upper left of each image) as differentiation in the iridocorneal angle is more dependant on the size of the fish than the absolute age.

Inspection of the iris, ciliary zone, and anterior segment angle in the mature zebrafish highlights the dramatic differentiation that has occurred from embryonic stages (Figure 6H–N). To investigate the differentiation process within the ocular angle, we analyzed histological sections from dorsal and ventral regions at various timepoints between embryonic and adult stages (Figure 7 and 8). During this protracted development, the iris has grown extensively and a defined non-pigment epithelium, continuous with the retinal neuroepithelium, is present by 20 dpf. The zebrafish iris stroma is not contractile and lacks muscle cells. In addition, zebrafish do not show ciliary processes and instead we refer to the region underlying the base of the iris as the ciliary zone. The iris stoma remains stratified throughout development, with the same ordering of pigment cells as in the embryo. However, in addition to the posterior iridophores and superficial xanthophores, melanophores are interspersed with non-pigmented cells throughout the stroma. These cells appear at approximately 5–7 dpf. In the adult, the iridophore layer can be further sub-divided with superficial cells having iridosomes arranged perpendicular to iridosomes from cells closer to the pigmented epithelium (Figure 7F, 8F). The iris stroma also contains non-uniform clusters of vascular vessels that are more dense at the base where the iris is thicker. Similar to mammals, the superficial surface of the iris stroma is devoid of an epithelial covering.

**Figure 9:**
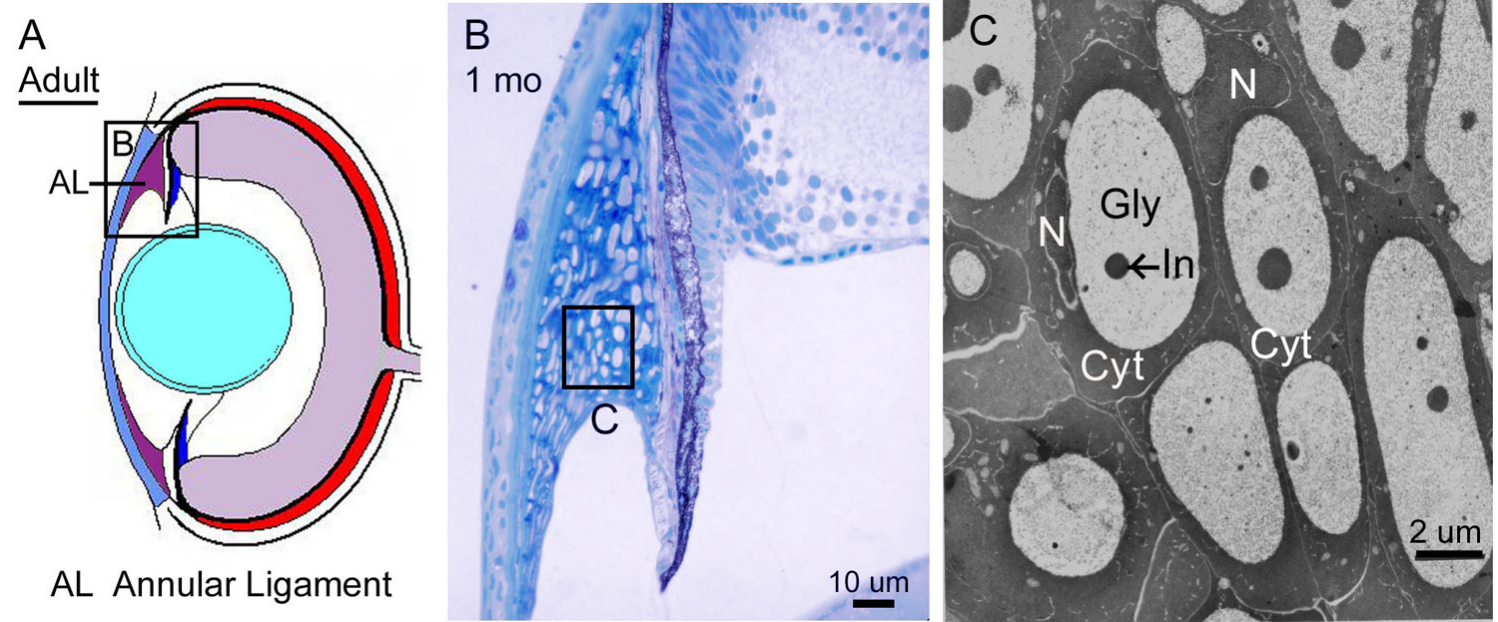
**Morphology of the annular ligament. **Diagram of an adult zebrafish eye shows the location of insets for morphological analysis of the annular ligament (A). Histological section through the dorsal iridocorneal angle with extensive annular ligament (B). TEM micrograph of annular ligament cells (C). Glycoprotein aggregates, Gly, nuclei, N, and cell cytoplasm, Cyt, are indicated. Arrow points to cytoplasmic inclusion, In, within the glycoprotein aggregates.

**Figure 10:**
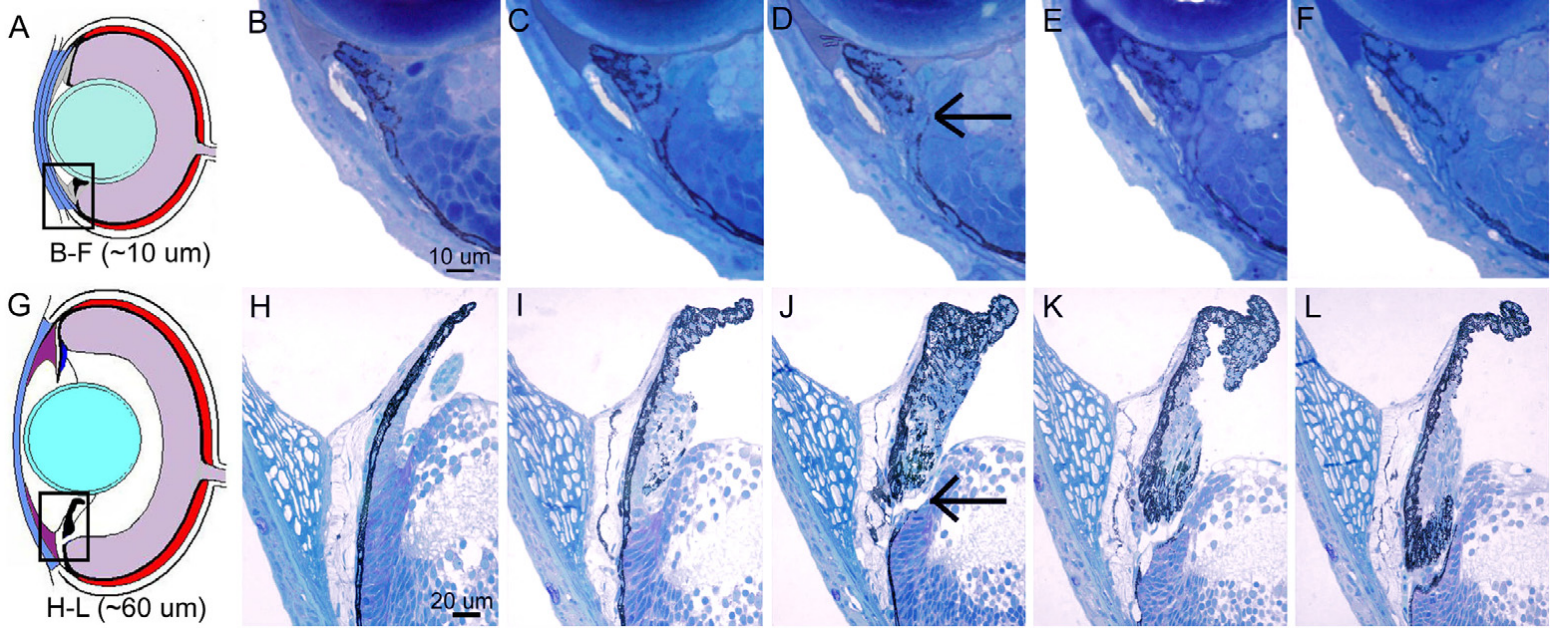
**Serial section analysis through the ventral angle. **Diagram of an embryonic (A) and mature (G) zebrafish eye shows the location of serial histological sections. Serial histological sections through a ~10 micron region of the 5 dpf embryonic ventral angle (B-F) and a ~60 micron region of the 1 month mature ventral angle. Note the elaboration of a canalicular sinus network. Arrows indicate the central region through the ciliary canal (D, J).

**Figure 11:**
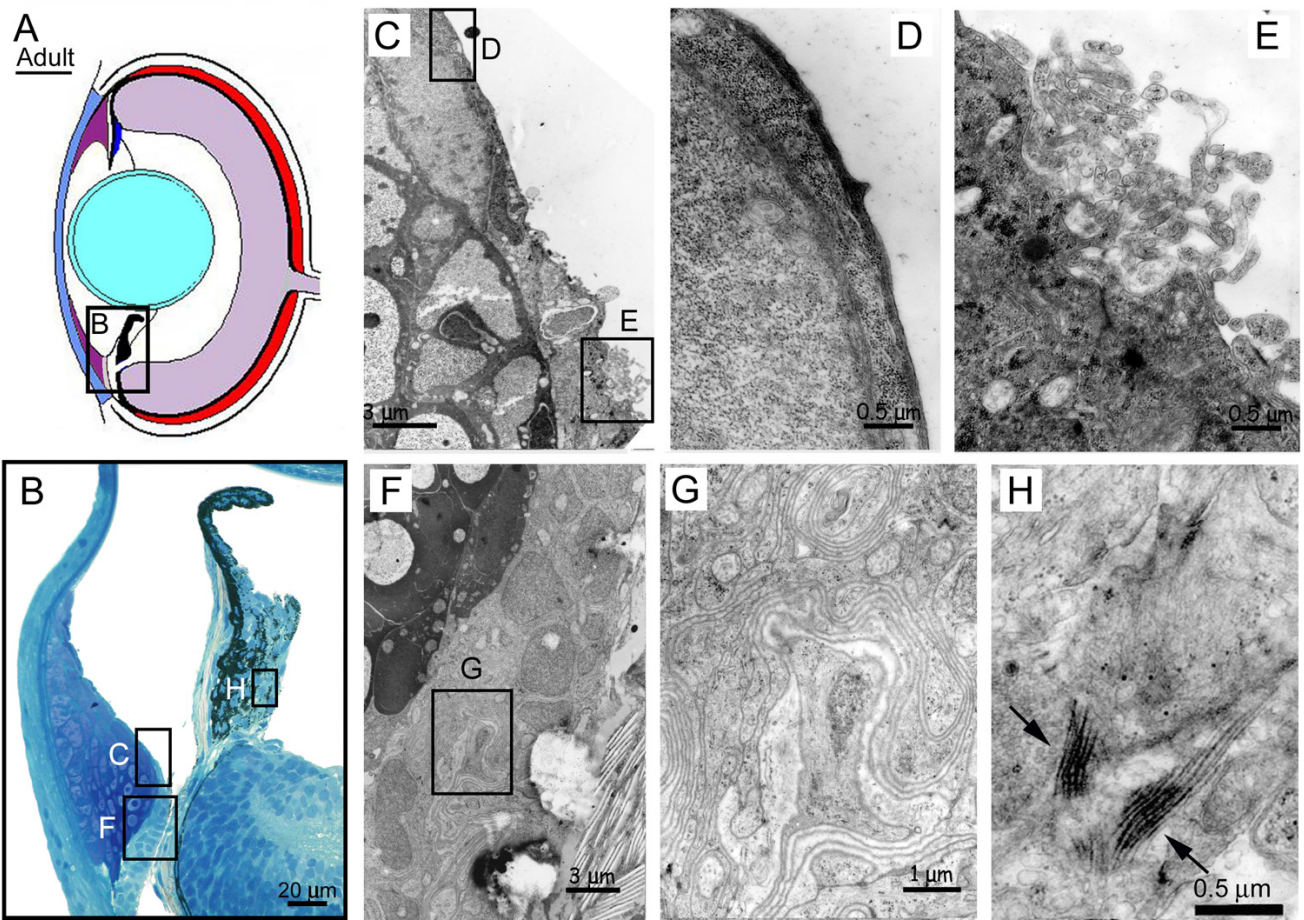
**Ultrastructure of ventral iridocorneal angle specializations. **Diagram of a mature (A) zebrafish eye shows the location of histological inset. Histology of ventral iridocorneal angle specializations in a 1 month old zebrafish (B). Low magnification TEM micrograph of endothelium lining annular ligament (C). High magnification TEM micrograph of smooth endothelium lining the central annular ligament (D). High magnification TEM micrograph of involuted and absorptive-appearing cells lining the iridocorneal angle (E). Low magnification TEM micrograph of mesothelium lining the iridocorneal canal (F). High magnification TEM micrograph of involuted and absorptive-appearing mesothelium lining the iridocorneal canal (G). High magnification TEM micrograph showing electron dense, thick filaments within myoepithelial cells of the ciliary "thumb" projection (arrows, H).

The ciliary zone provides a site for lens zonule attachment, and in mammals and avians, this is also the site of aqueous humor production. In zebrafish, electron microscopy revealed differences in ciliary zone non-pigmented epithelial cells between the dorsal versus ventral regions. Non-pigmented epithelium in the dorsal ciliary zone is thicker than that in the ventral region (Figure 6J, K, M, N). Dorsal non-pigented ciliary epithelium is highly convoluted and secretory in appearance (Figure 6J, K). These cells are enriched in cytoplasmic extensions, golgi apparatus, and intracellular vesicles. The dorsal ciliary epithelium is ultrastructurally very similar to the non-pigmented epithelium lining ciliary processes of mammals [17]. In the ventral region, the non-pigmented epithelium is also secretory in appearance, but contains less cytoplasmic extensions and intracellular vesicles (Figure 6M, N). In the extreme ventral region, where there are canalicular specializations at the angle, the non-pigmented epithelium is absent (Figure 10, 11).

### Anterior chamber angle and annular ligament

A striking difference between the embryonic angle and that of the adult zebrafish, is the presence of the annular ligament. The annular ligament is a prominent, but highly variable feature between species of bony fish [1,18]. The annular ligament is named for its ligament-like, fibrous meshwork appearance in aldehyde-fixed preparations. This meshwork fills much of the iridocorneal angle and runs circumferentially (thus annular) throughout the anterior segment. In zebrafish, this structure is visible by histology at approximately 17 dpf (Figure 7 and 8). It appears to differentiate from the mass of mesenchymal cells that are present during early developmental stages in the angle region. The shape and extent of the annular ligament varies greatly between dorsal and ventral regions of the eye. In the dorsal region, the inner surface of the annular ligament forms a deep "U" shape (Figure 7F). In the ventral region, the extent of the annular ligament is reduced and forms a "funnel" shape (Figure 8F). As part of this shape difference of the anterior segment angle, the iris projects slightly forward in the dorsal region, while in the ventral half, the iris bends posteriorly (Figure 9A). By histology, the annular ligament appears porous and devoid of nuclei. However, electron microscopy revealed that small, irregularly shaped nuclei are present in annular ligament cells (Figure 9C). Ultrastructurally these cells contain very large accumulations of non-membrane bound aggregates of what appears to be glycoprotein. In other teleosts, these cells have been described as glycogen aggregates [18], but in zebrafish, the fibrilar nature of the aggregated material appears to be glycoproteinatious in nature. Interspersed within the glycoprotein aggregates are islands of darker staining cytoplasm. The surface of the annular ligament facing the anterior chamber is lined by an endothelium. The endothelium, at the base of the annular ligament, becomes more villous with numerous cytoplasmic convolutions. The annular ligament itself thins at the edges and extends onto both the posterior surface of the cornea and the anterior surface of the iris at its base.

### Ventral angle specializations

Serial histological sectioning in both embryos and adults revealed specializations at the iridocorneal angle within a narrow region of the ventral anterior segment (Figure 10). These specializations include a network of canals and openings that lead to episcleral vasculature. Within this region, the iris and ciliary zone dramatically change in morphology. The tip of the iris forms a nodule and bends sharply towards the vitreous (Figure 10I–L). Another feature of this ventral specialization is the loss of the non-pigmented ciliary epithelium. At the ciliary zone, the iris thickens and forms a thumb-like structure that projects into the vitreous and contains melanophores and non-pigmented myoepithelial cells (Figure 10I, 11B). By electron microscopy, the non-pigmented myoepithelial cells show thickened, electron dense intracellular filaments and may have contractile function to regulate lens position via the zonules (Figure 11H). A ventral canal is present as early as 3 dpf and matures significantly into a canalicular network as the embryo grows. This complex structure appears to arise as a specialization of the embryonic fissure.

**Figure 12:**
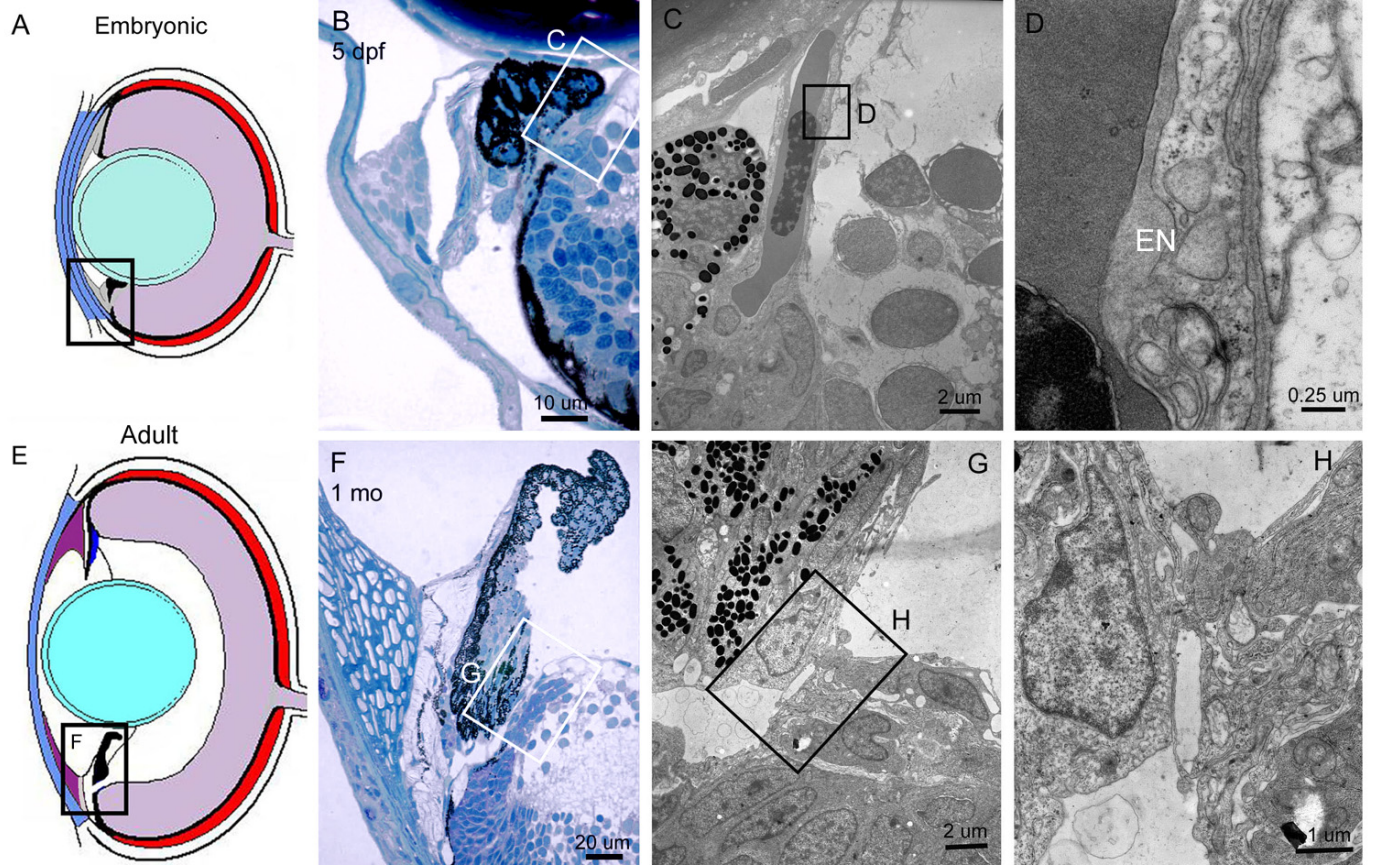
**Ultrastructure of ventral ciliary canal. **Diagrams of embryonic (A) and adult (E) zebrafish eye showing the locations of histological insets. Histological section through the central portion of the ventral ciliary canal (B). Low magnification TEM micrograph showing differentiating endothelial cells associated with the sinus (C). High magnification TEM micrograph of the undifferentiated endothelial cell, EN (D). Histological section through the central portion of the ventral ciliary canal (F). Low magnification TEM micrograph showing mature endothelial cells overlying the sinus (G). High magnification TEM micrograph of the mature endothelial cells (H). Note the cytoplasmic extensions and presence of large intracellular vacuoles.

Within this highly differentiated ventral region, the iris stroma has an expanded layer of non-pigmented mesothelial cells that form a band of tissue separating the annular ligament from the iris surface (Figure 11B, inset 11F). In young specimens this region is prone to separation during histological preparation, suggesting a loose extracellular matrix (for example Figure 12B). By 17 dpf, a distinct boundary is visible within the central part of this strip of mesenchymal cells. In the adult, this boundary develops into a channel that connects to an angular aqueous plexus and represents a major branch of the canalicular network (Figure 11B). The angular aqueous plexus is external to the iridocorneal angle, adjacent to the sclera, and in close proximity with connections to the vascular choroid. The mesothelial cells that line this canalicular network at the iridocorneal angle are highly involuted with whirls of internal membranes (Figure 11G). Ultrastructurally, cross-sections of these cells resemble absorptive intestinal epithelia (Figure 11E).

Within this defined ventral region at the ciliary zone, there is another major branch of the canal that penetrates through the base of the iris and also connects to the angular aqueous plexus (Figure 12). Similar to the canal at the iridocorneal angle, this passage is also endothelial lined. At 5 dpf, flattened endothelial cells line the narrow opening which occasionally contains undifferentiated and red blood cells (Figure 12C, D). In the mature fish, the endothelial cells that line this ciliary canal have further differentiated (Figure 12G). These cells line the opening to the canal and show large vacuoles and extensive cyctoplasmic extensions (Figure 12H). The dorsal angle completely lacks this canalicular network and does not have either the band of absorptive-looking tissue at the iridocorneal angle or the subjacent ciliary passage.

## Discussion

### Zebrafish as a model for anterior segment development and disease

Zebrafish have several experimental advantages that have made this a valuable model organism for analysis of mechanisms of development and disease. Experimental advantages include the ability to efficiently conduct mutational analyses, including those designed to reveal complex genetic interactions. Ease of transgenesis facilitates gain-of-function and cell labeling studies. Cell labeling coupled with rapid, transparent, and external embryogenesis enable in vivo cell behavior studies during development. In our analysis, we have characterized the morphogenesis of the zebrafish anterior segment of the eye in order to establish an overview reference for future studies on the mechanisms of development and diseases of this structure. Ontogeny of the zebrafish anterior segment is protracted occurring over the first month of development. Some structures of the anterior segment such as the lens and cornea differentiate relatively early and show only peripheral growth. Other structures such as the iris, ciliary zone, and iridocorneal angle undergo dramatic morphological changes during this time. Our analysis of zebrafish anterior segment development has also identified differences between the dorsal and ventral regions. In particular, structures specialized for regulating aqueous humor dynamics are localized in a dorsoventral specific manner.

### Comparing anterior segment development in zebrafish and mammals

Overall morphogenesis of the anterior segment in zebrafish is similar to mammals and other vertebrates but there are some differences. For example the lens, although derived from surface ectoderm, delaminates as opposed to invaginating. Periocular mesenchyme appears to contribute to the corneal endothelium, iris stroma and angle specializations like other vertebrates. However, there appears to be a proportionally smaller bolus of immigrating mesenchymal cells and migration does not occur in obvious waves as in the chick [4,19]. Local proliferation appears to be a driving force for growth of the anterior segment in zebrafish, similar to that in the mouse [6,20]. The relative timing of differentiation for the various components of the zebrafish anterior segment is like that for other vertebrates, suggesting conservation in tissue interaction and inductive events [19,21].

While hallmarks of anterior segment development in zebrafish appear to be largely conserved with other vertebrates, anatomical specializations exist. For example, the iris stroma is non-contractile and devoid of muscle cells and the ciliary "body" does not contain processes or a circumferential band of muscle. For this reason, we refer to the region adjacent and posterior to the iris as the ciliary zone, and not the ciliary body. The ciliary zone, however, is expanded and has a morphology consistent with contraction in a small area of the ventral-most region. The ciliary epithelium also displays dorsoventral differences. The ciliary epithelium in the dorsal eye appears specialized for secretion of aqueous humor while the ciliary epithelium in the ventral most region does not show extensive ultrastructural specializations. Instead, the ventral ciliary zone appears specialized for aqueous humor drainage. Other anatomical differences with mammals can be found within the iris. Within the iris stroma, in the place of muscle cells are lamina of pigment cells of various types. These include melanophores, iridophores, and xanthophores. The exact function of these chromophores in the iris are unknown, but they likely serve to prevent light from entering the eye in regions outside of the pupil. Finally, similar to the mammalian iris, the zebrafish iris is highly vascularized (Figure 5 and 6).

### Morphological specializations at the iridocorneal angle

Another difference with mammals is found at the iridocorneal angle. In mammals, the angle is lined circumferentially with a trabecular meshwork, a complex structure of extracellular "trabecular" beams and a "meshwork" of absorptive endothelial cells. The trabecular meshwork forms a resistant barrier for aqueous humor outflow and covers Schlemm's canal. Schlemm's canal is a network of small canalicular passages leading to episcleral vessels where aqueous humor is ultimately drained. In zebrafish, the iridocorneal angle is lined with the annular ligament. This structure does not appear to be functionally analogous to the trabecular meshwork of mammals. However, within the ventral region of the angle, a specialized canalicular network of endothelial-lined openings does appear to be functionally analogous to the aqueous humor outflow structures of mammals. The circumferential ring of villous endothelial cells overlying the annular ligament at the iridocorneal angle may also function in absorption of metabolic byproducts, ions, or proteins in the aqueous humor. The anatomy of the mature zebrafish anterior segment suggests that in this species aqueous humor is produced primarily by dorsal ciliary epithelial cells, flows around the iris and into the anterior chamber, and then exits via a canalicular network in the ventral-most region. Additional experiments will be required to investigate this possibility further.

## Conclusion

Cumulatively, our anatomical findings provide a reference point for utilizing zebrafish in genetic studies of the mechanisms of development and maintenance of the anterior segment. We find that while the morphological details of anterior segment structures in the zebrafish differ from higher vertebrates in many aspects, these structures show an overall conservation in anatomy and develop from similar cellular lineages. As in other vertebrates, surface ectoderm, periocular mesenchyme, and the anterior margin of the retinal neuroectoderm contribute to structures of the anterior segment. The timing of morphogenesis is also conserved among vertebrates. For example the lens and cornea are first to mature, while the iridocorneal angle does not reach adult morphology until later stages. In addition to providing a baseline for genetic studies of anterior segment development, our observations coupled with previous physiological approaches [22], provide insights into how aqueous humor is regulated in zebrafish and support the use of this species for investigation of anterior segment development and disease.

## Methods

### Specimens

Wild type zebrafish (*Danio rerio*) of the AB/AB and TL/TL backgrounds were reared under standard conditions with a light cycle of 14 h light/10 h dark [23]. No differences in anterior segment anatomy were observed between these two stains. Specimens were collected at various times of development from 24 hours to 2 months post fertilization. All individuals were photographed to document the body length prior to histological analysis. Prior to fixation, fish were anesthetized in 0.2 mg per ml of ethyl 3-aminobenzoate methanesulfonate (tricane). All experiments were performed in compliance with the ARVO statement for use of animals in vision research.

### Light microscopy

Fish were fixed in primary fixative [2% paraformaldehyde, 2.5% glutaraldehyde, 3% sucrose, 0.06% phosphate buffer (pH 7.4)] at 4°C for at least 24 hours. Fish were washed in 0.1 M phosphate-buffered saline (PBS), dehydrated through an ethanol series and propylene oxide and then infiltrated with EMbed-812/Araldyte resin mixture. Traverse, semi-thin (1 μm), plastic sections were cut with a glass knife on a JB4 microtome. Serial sections were collected from the central retina, defined as maximum lens diameter at the optic nerve. Following heat fixation to glass slides, sections were stained with 1% Toluidine Blue in 1% Borax buffer. Images were captured using a Nikon coolpix 995 digital color digital camera mounted on a Nikon E800 compound microscope with a 60X oil-emersion objective.

### Electron microscopy

Fish were fixed in primary fixative and washed as for LM. Specimens were then post-fixed with 1% Osmium Tetroxide on ice for 1 hour to preserve membranes. Fish were dehydrated through a methanol series and acetonitrile and infiltrated with EMbed-812/Araldyte resin mixture. Ultrathin sections (60–70 nm) were collected on coated grids and stained with uranyl acetate and lead citrate for contrast. Images were captured using Hitachi H600 TEM.

## List of abreviations

TEM: transmission electron microscopy

hpf: hours post fertilization

dpf: days post fertilization

## Authors' contributions

KS collected and processed all specimens for morphological analysis and helped to draft the manuscript. BL conceived of the study, participated in its design, coordination, and data interpretation, and helped to draft the manuscript. Both authors read and approved the final manuscript.
